# The Information Void in Asymptomatic Chronic Disease: A Digital Health Framework for Understanding Social Media Health Information Seeking in Young Adults

**DOI:** 10.2196/86489

**Published:** 2026-04-07

**Authors:** Victoria Sze Min Ekstrom

**Affiliations:** 1 Department of Gastroenterology and Hepatology, Singapore General Hospital, 20 College RdSingapore, 169856, Singapore, 65 63214684; 2 SingHealth Duke-NUS Medical Humanities Institute, Singapore

**Keywords:** digital health, social media, health information seeking, asymptomatic chronic disease, prediabetes, dyslipidemia, hypertension, fatty liver disease, nonalcoholic fatty liver disease, NAFLD, young adults, digital health literacy, health literacy, behavior change, patient engagement, misinformation, TikTok, infodemiology

## Abstract

Nearly 1 in 4 young adults has a chronic condition, yet many feel well despite their diagnosis. Asymptomatic conditions such as prediabetes and hypertension create a unique vulnerability to digital health misinformation, particularly on platforms where inaccurate content is prevalent. Conventional clinical responses, which often just warn patients about online misinformation, fail to address the underlying drivers of this behavior. This viewpoint proposes a novel disease characteristic–based vulnerability framework to understand this challenge, grounded in established behavioral science theories such as the capability, opportunity, and motivation–behavior model; temporal discounting; and the concept of information voids in infodemiology. We identify a critical “information void” for asymptomatic conditions managed primarily through lifestyle modification. This void, created by the absence of symptomatic feedback combined with delayed clinical biomarker feedback, compels patients to seek information online. Instead of viewing this information seeking as a problematic deviation, we reframe it as a “digital phenotype” indicating a patient’s readiness for behavior change. Through case studies illustrating how this framework applies to specific conditions (prediabetes, nonalcoholic fatty liver disease, and untreated hypertension), we demonstrate its practical utility for clinicians, health systems, and policymakers. Evidence supports a multipronged approach: integrating digital health literacy into clinical encounters, providing curated evidence-based resources, and pursuing strategic institutional engagement in digital spaces. While acknowledging the framework’s deliberate simplification and the need for culturally sensitive adaptation across diverse health care settings, this viewpoint offers a generalizable strategy for engaging with patients’ information needs, helping transform a public health challenge into an opportunity for empowerment.

## Introduction

Asymptomatic metabolic and cardiovascular conditions are increasingly prevalent among young adults, creating a growing public health concern. Prediabetes now affects approximately 24% of this demographic [1], the prevalence of nonalcoholic fatty liver disease has nearly doubled over 2 decades [2], and 22.4% of adults aged 18 to 39 years have hypertension [3]. Initial management of these conditions typically emphasizes lifestyle modification and relies on biomarker reassessment months later. This approach creates a prolonged “information void” where patients feel healthy but lack actionable guidance. To fill this void, young adults increasingly turn to digital platforms, with scoping reviews confirming their primary use of search engines and social media for health information [4]. For example, a recent survey found that 65.5% of young women intentionally seek health advice on TikTok [5]. However, these platforms contain widespread misinformation. One systematic review found misinformation rates of around 40% for noncommunicable diseases [6], while topic-specific analyses show that less than half of educational videos from influencers may be factual [7] and more than half of popular attention-deficit/hyperactivity disorder content is misleading [8].

This convergence of rising disease prevalence and digital information seeking creates a significant challenge for evidence-based care. Clinical guidelines often formalize the long intervals between encounters that define the information void. For instance, recommendations include lipid monitoring every 3 to 12 months [9] or repeat diabetes screening every 3 years for normal results [10]. While the individual components of this problem are recognized, a comprehensive explanatory framework is missing. There is no model that links specific disease characteristics, such as asymptomatic presentation and delayed, abstract feedback, to a differential vulnerability to health misinformation in young adults. Consequently, conventional clinical advice to simply “be careful online” fails to address the underlying drivers of this vulnerability or offer practical, evidence-based alternatives.

The need to address this gap is urgent. The rising epidemiologic burden is substantial, with 27.1% of young adults now living with multiple chronic conditions [11]. This trend intersects with their established digital health information–seeking behaviors [4,5]. Failing to intervene has serious, long-term consequences. Asymptomatic conditions diagnosed in young adulthood are not benign. Early hypertension is associated with a significantly higher risk of later cardiovascular events [12] and adverse late-life neuroimaging biomarkers [13]. Furthermore, prediabetes in this population progresses to diabetes in 9.5% of cases within 5 years [14]. To mitigate these risks, it is critical to develop targeted strategies that fill the information void with credible guidance.

This article has 3 objectives: First, we synthesize evidence from infodemiology, behavioral science, and digital health to construct a disease characteristic–based framework that explains why patients with certain conditions are disproportionately vulnerable to online misinformation. Second, we apply this framework through illustrative case studies to demonstrate its practical utility. Third, we propose evidence-based digital health interventions designed to address this vulnerability. Our intended audience encompasses frontline clinicians who encounter these patients daily, health systems and policymakers seeking scalable strategies, and researchers working at the intersection of digital health and chronic disease prevention.

## Evidence and Theoretical Foundations

### Digital Health Information Seeking in Chronic Disease Management

A growing body of evidence documents the role of digital platforms in shaping health behaviors among individuals with chronic conditions. A systematic review by Zhao and Zhang [15] found that social media platforms serve as important sources of both informational and emotional support for individuals managing chronic diseases, with users actively seeking peer experiences and practical self-management strategies. Importantly, the type of platform used and the quality of the information found vary considerably by condition. For highly symptomatic conditions with visible communities (such as diabetes or inflammatory bowel disease), patients often find structured peer support forums and professionally curated content [16]. In contrast, for asymptomatic or preclinical conditions such as prediabetes and early-stage fatty liver disease, the digital landscape is far less organized, with information seekers encountering a fragmented mix of commercial wellness content, personal testimonials, and algorithmically amplified claims [17].

This disparity in digital information quality has measurable consequences. A cross-sectional analysis of TikTok content related to nonalcoholic fatty liver disease found that most popular videos were produced by nonmedical content creators and contained significant inaccuracies regarding dietary recommendations and disease reversibility [18]. Similarly, studies evaluating diabetes and metabolic health information on TikTok have found low reliability and quality scores, with a predominance of commercially motivated messaging from noncredentialed health coaches and content that lacks actionable, evidence-based guidance [19]. These findings are not limited to Western platforms; analyses of Weibo and WeChat health content in China have identified comparable patterns of misinformation in metabolic disease discussions, suggesting that the phenomenon transcends specific cultural or platform contexts [20].

### Theoretical Underpinnings

Our proposed framework draws on several established theoretical models. First, it is grounded in the concept of “data voids” and “information voids” from infodemiology, which describes how gaps in authoritative information create vacuums that are filled by low-quality or misleading content [21,22]. The work by Golebiewski and Boyd [21] on data voids in search engines demonstrates that, when reliable information is scarce for a given query, algorithmically ranked results disproportionately surface fringe or commercially motivated content. We extend this concept to the clinical domain, arguing that the temporal gap between diagnosis and biomarker feedback creates a functionally analogous information void for patients.

Second, the framework builds on the Behavior Change Wheel (capability, opportunity, and motivation–behavior; COM-B) model, which posits that behavior arises from the interaction of capability, opportunity, and motivation [23]. From this lens, the information void represents a deficit in both psychological capability (knowledge of how to implement lifestyle changes) and environmental opportunity (access to credible, actionable resources). The patient’s subsequent turn to social media is a motivated attempt to address these deficits, which the COM-B model would predict.

Third, we draw on temporal discounting theory from behavioral economics [24,25]. This well-established phenomenon whereby individuals systematically undervalue delayed rewards relative to immediate ones is particularly relevant to asymptomatic conditions. When the clinical message is essentially “change your behavior now to prevent a disease outcome in 10 to 30 years,” temporal discounting predicts low motivational salience in the absence of proximate feedback. Social media content creators exploit this cognitive bias by offering promises of rapid, tangible results (eg, “reverse your prediabetes in 30 days”), providing the short-term validation that is absent from long-horizon prevention messages.

Finally, uses and gratifications theory [26] provides a complementary perspective, explaining why young adults actively choose social media over clinical sources. This theory posits that individuals are active agents who select media to satisfy specific needs, including informational needs, social identity needs, and emotional validation needs. For a young adult newly diagnosed with an asymptomatic condition, social media uniquely satisfies all 3: it provides practical “how-to” information, connects the individual with a peer community that shares their experience, and offers emotional reassurance through success narratives. Traditional clinical encounters, constrained by time and format, often address only the informational need and may not even do so adequately for lifestyle-focused conditions.

## The Information Void Framework: Disease Characteristics as Vulnerability Determinants

Building on the theoretical foundations outlined above, our proposed disease characteristic–based framework provides a structured explanation for why certain patient populations are disproportionately vulnerable to digital health misinformation as referenced in Table 1. The framework classifies chronic conditions along two axes: (1) symptom presence (whether the condition produces noticeable symptoms that provide ongoing sensory feedback to the patient) and (2) primary management strategy (whether initial management relies primarily on pharmaceutical intervention, which introduces its own structured feedback loop, or on lifestyle modification, which does not). The intersection of these axes generates 4 distinct quadrants, each characterized by a unique feedback environment and a corresponding level of vulnerability to online misinformation.

**Table 1.** Disease characteristic–based framework for vulnerability to digital health misinformation.

	Symptomatic		Asymptomatic	
	Pharmaceutical (quadrant 1)	Lifestyle (quadrant 2)	Pharmaceutical (quadrant 3)	Lifestyle (quadrant 4)
Examples	Type 1 diabetes, rheumatoid arthritis, and epilepsy	Symptomatic IBS^a^ (dietary) and chronic pain (exercise based)	Treated hypertension and statin-managed dyslipidemia	Prediabetes, untreated hypertension, NAFLD^b^, and early dyslipidemia
Symptom feedback	Present: symptoms validate management (eg, glucose swings)	Present: symptoms provide direct feedback on lifestyle changes	Absent: no somatic cues	Absent: no somatic cues; the patient feels well
Clinical feedback	Rapid and structured: daily glucose monitoring and dose titration	Moderate: symptom diaries and functional improvement over weeks	Structured but abstract: periodic biomarkers (eg, BP^c^ readings and lipid panels) linked to medication adjustments	Delayed and abstract: biomarker reassessment months later with no medication anchor; feedback disconnected from daily effort
Information void	Minimal: multiple feedback sources reduce the need for external validation	Low-moderate: symptom changes provide some validation of effort	Moderate: medication provides a structured anchor, but the lack of symptoms reduces urgency for additional information seeking	Maximal: no symptoms, no medication anchor, and no proximate feedback; the patient is left with broad lifestyle advice and months of uncertainty
Misinformation vulnerability	Low	Low-moderate	Moderate	High

a IBS: irritable bowel syndrome.

b NAFLD: nonalcoholic fatty liver disease.

c BP: blood pressure.

We define “vulnerability to digital health misinformation” in this context as the degree to which a patient’s clinical situation creates conditions that predispose them to seek, encounter, and potentially act upon inaccurate health information online. This vulnerability is not a fixed individual trait but rather an emergent property of the interaction among disease characteristics, the clinical feedback environment, and the digital information landscape. It is highest when three conditions converge: (1) the patient lacks somatic feedback (no symptoms to confirm or disconfirm advice), (2) clinical feedback is infrequent or abstract (delayed biomarker results rather than immediate, tangible changes), and (3) the information void created by these first 2 factors intersects with a digital ecosystem where low-quality content is algorithmically amplified.

The critical distinction between quadrants 3 and 4 warrants further elaboration. In quadrant 3 (asymptomatic and pharmaceutically managed), conditions such as statin-treated dyslipidemia involve a structured feedback loop: the patient takes a medication daily, attends periodic follow-up appointments, and receives biomarker results (eg, low-density lipoprotein cholesterol levels) that are directly linked to medication dose adjustments. Although the patient feels no symptoms, the medication itself serves as a tangible, daily anchor to the management plan. The act of taking a pill is a concrete behavior with a clear link to the clinical goal, and adjustments to that regimen provide relatively prompt clinical feedback. In quadrant 4, in contrast, no such anchor exists. The patient with prediabetes managed through lifestyle modification alone is told to “eat better and exercise more” and then returns months later for a repeat hemoglobin A_1c_ test. During those intervening months, there is no structured touchpoint, no daily behavioral anchor linked to the clinical plan, and no proximate feedback mechanism to confirm whether their efforts are succeeding. It is precisely this absence of any feedback loop—somatic, pharmacological, or clinical—that creates the maximal information void and drives patients to seek validation and guidance from digital sources.

We acknowledge that this 2×2 framework is a deliberate simplification of a complex reality. Chronic conditions do not always fit neatly into 1 quadrant; many evolve over time (prediabetes progressing to treated diabetes moves from quadrant 4 to quadrant 3 or 1), and individual patients may occupy different positions based on their specific treatment regimen, comorbidities, and health care context. Moreover, the level of vulnerability is modulated by factors not captured in the framework itself, including health literacy, cultural context, socioeconomic status, health care system structure, and the availability of community-based support [27,28]. A patient with prediabetes in a resource-rich setting with regular access to a multidisciplinary care team faces a very different information void from that of a patient in a resource-limited setting without such support. We present the framework not as a comprehensive model of all factors influencing misinformation vulnerability but as a parsimonious heuristic that isolates 2 modifiable, disease-level characteristics with clear implications for clinical practice and health system design.

## Applying the Framework: Illustrative Case Studies

To demonstrate the practical utility of the framework, we present 3 illustrative case studies (Textboxes1^3^) showing how the information void manifests differently across quadrant 4 conditions and how it maps onto specific types of misinformation encountered on social media platforms.

Textbox 1. **Case study 1: prediabetes and “reverse your diabetes” content on TikTok.**

- **Clinical scenario:** an individual aged 28 y is diagnosed with prediabetes (hemoglobin A_1c_ [HbA_1c_]=6.1%) at a routine checkup. She feels completely well. Her clinician advises dietary modification and increased physical activity, with a repeat HbA_1c_ measurement in 6 months.
- **Information void:** for the next 6 months, this patient has no clinical feedback whatsoever. She has no symptoms to monitor, no medication regimen to anchor her daily behavior, and no way to know whether her dietary changes are “working.”
- **Digital misinformation pathway:** she searches TikTok for “how to reverse prediabetes.” The algorithm surfaces high-engagement videos promising “reverse your prediabetes in 30 days with this one supplement” and “the food your doctor won’t tell you about.” These videos exploit temporal discounting by promising rapid results and fill the information void with concrete (if unfounded) action plans. The patient purchases an unregulated supplement and follows an elimination diet with no evidence base.
- **Framework application:** the framework predicts this trajectory. Quadrant 4 conditions (asymptomatic + lifestyle managed) create maximal vulnerability because the combination of no somatic feedback and delayed clinical feedback leaves the patient entirely reliant on external sources for validation that their efforts are worthwhile.

Textbox 2. **Case study 2: nonalcoholic fatty liver disease (NAFLD) and “liver detox” misinformation.**

- **Clinical scenario:** a man aged 32 y is incidentally found to have hepatic steatosis on abdominal ultrasound during workup for another issue. He has mildly elevated alanine aminotransferase. He is advised to lose weight through diet and exercise, with repeat liver function tests and imaging in 12 months.
- **Information void:** the patient has no symptoms and no medication and faces a 12-month wait for clinical feedback. He may not even fully understand what “fatty liver” means or how serious it could become.
- **Digital misinformation pathway:** searching “fatty liver cure” on social media yields a flood of “liver detox” products, “liver cleanse” juice protocols, and testimonials claiming dramatic improvement from unproven supplements. The NAFLD information landscape is particularly prone to commercial exploitation because the term “liver detox” is already embedded in wellness culture, creating a preexisting market for products targeting health-anxious consumers [29].
- **Framework application:** NAFLD exemplifies quadrant 4 vulnerability in its most extreme form—an exceptionally long feedback interval (often 6-12 months), a condition name that is poorly understood by the public, and a commercial misinformation ecosystem that is already well established around “liver health.”

Textbox 3. **Case study 3: untreated hypertension and supplement-based “natural” blood pressure (BP) cures.**

- **Clinical scenario:** an individual aged 25 y has stage 1 hypertension (BP of 135/85 mm Hg) identified at a routine visit. Given his age, low cardiovascular risk score, and absence of target organ damage, his physician recommends a trial of lifestyle modification (sodium restriction, Dietary Approaches to Stop Hypertension diet, and exercise) before initiating medication, with follow-up in 3 months.
- **Information void:** unlike patients initiated on antihypertensive medication (quadrant 3), this patient has no pharmacological anchor. He may or may not own a home BP monitor. If he does, the natural variability in readings may itself become a source of anxiety and confusion.
- **Digital misinformation pathway:** searching “lower blood pressure naturally” yields content promoting magnesium supplements, beet juice “cures,” and breathing techniques with exaggerated efficacy claims. Some content explicitly frames medication as harmful, reinforcing the patient’s desire to avoid pharmaceuticals and potentially delaying necessary treatment if lifestyle modification proves insufficient.
- **Framework application:** this case illustrates how quadrant 4 vulnerability can have particularly serious consequences when it delays transition to quadrant 3 (pharmaceutical management). The information void does not just expose patients to misinformation—it can actively impede appropriate escalation of care.

## Reframing Information Seeking as a Clinical Signal: The Digital Phenotype Concept

In response to the challenge described by the framework, we advocate for a fundamental shift in clinical perspective: reframing active online information seeking from a problematic behavior to a valuable clinical signal, which we term the “digital phenotype.” This reconceptualization is firmly grounded in the COM-B model described above [23,30]. From this perspective, a patient who actively searches for information about their new diagnosis is demonstrating psychological capability (understanding the diagnosis), motivation (a desire to act), and an attempt to create opportunity (finding resources for self-management). This proactive behavior maps directly onto theoretical domains framework domains such as “social influences” and “environmental context and resources,” which are critical determinants of health behavior but are often underaddressed in time-constrained clinical encounters. Instead of viewing this as noncompliance or a challenge to medical authority, clinicians can interpret it as a positive prognostic indicator.

This concept finds support in a growing body of literature that links online health information seeking with positive health indicators, such as higher patient activation levels, more appropriate use of health services, and greater vaccine uptake [29,31,32]. However, the digital phenotype is a nuanced signal that requires careful interpretation. The same behavior can also stem from confusion, anxiety, or low digital health literacy, which may predispose individuals to seek out and trust unreliable sources [33,34]. Furthermore, some studies have shown that general online searching does not always correlate with adherence to specific clinical recommendations such as cancer screening guidelines [35]. Therefore, the clinical utility of the digital phenotype lies not in a simple binary assessment but in its function as a triage tool. A brief, nonjudgmental inquiry at diagnosis—such as “Many people look for information online after a new diagnosis. Have you found anything helpful or confusing?”—can open a dialogue. This allows clinicians to stratify support, offering foundational digital literacy skills to those who seem overwhelmed or misinformed while providing curated, high-quality resources to those who are already motivated and capable, thereby channeling their proactive energy toward evidence-based pathways.

## Evidence-Based Digital Health Interventions

The evidence base supports a multipronged intervention strategy to constructively engage this digital phenotype and fill the information void. The first pillar of this strategy is the direct provision of digital health literacy education. This approach is backed by high-level evidence; a 2023 systematic review and meta-analysis of digital interventions for chronic diseases found a large and statistically significant improvement in eHealth literacy (standardized mean difference=1.22) [33]. Other meta-analyses have corroborated these findings, demonstrating that health literacy interventions can also lead to improvements in health status and self-efficacy [36]. Importantly, these reviews highlight that the most effective interventions are not one-off educational sessions but, rather, those that involve sustained engagement, use appropriate tools, and are integrated with primary care. The feasibility of this approach in the target population has been demonstrated in a recent pilot study of the Get Health‘e’ intervention, which successfully increased digital health knowledge and confidence among young adults [37]. Real-world implementation of such approaches is already emerging; for example, the Sun Life-KKH LITE Programme in Singapore provides a multidisciplinary, digitally delivered lifestyle intervention for children and families managing obesity integrating virtual coaching, peer WhatsApp communities, and structured physical activity sessions [38].

The second pillar involves the creation and dissemination of curated, developmentally aligned digital resources. It is not enough to teach patients how to evaluate information; health systems must also actively contribute high-quality, engaging content to the digital ecosystem. Currently, a significant gap exists. While medical professionals produce more accurate content, they struggle to compete with the reach and visibility of nonmedical influencers, who often have commercial incentives to promote unproven products or services [6]. This challenge is compounded by platform-specific algorithmic dynamics. On TikTok and similar short-form video platforms, recommendation algorithms prioritize engagement metrics (watch time, shares, and comments) over content accuracy [39]. This creates a structural disadvantage for evidence-based content, which tends to be more nuanced and less sensational than misinformation. Content from high–follower count influencers receives preferential algorithmic amplification regardless of its accuracy, creating a feedback loop in which misleading health claims gain visibility precisely because they are designed to maximize engagement [40]. To close this gap, health care organizations must create content that is not only evidence-based but also platform-native, leveraging formats that resonate with young adults, such as short-form video, and addressing the needs that drive them to these platforms: peer-style success narratives, practical tips, and emotional validation [5].

The third pillar is strategic institutional engagement in digital spaces. The current approach, which largely relies on the ad hoc efforts of individual physicians, is insufficient to counter the tide of coordinated, commercially driven misinformation. Many health care professionals already use social media for professional purposes, including patient education, and many report that it influences their own clinical perceptions and habits [41,42]. A coordinated strategy led by health care systems or professional societies could provide the resources, editorial oversight, and consistent branding necessary to build a trusted presence online. Critically, such strategies must also engage with platform governance. This could involve advocating for algorithmic transparency in health content curation, collaborating with platforms on content-labeling systems that distinguish evidence-based information from user-generated testimonials, and partnering with platform-native creators who have established credibility with young adult audiences to coproduce content that is both engaging and accurate [43]. Without addressing the structural and algorithmic factors that disadvantage evidence-based content, even the highest-quality clinical material will struggle to reach its intended audience.

## Research Priorities and Implementation Challenges

The implications of this framework are far-reaching, demanding action from clinicians, health systems, and digital platforms alike. For frontline clinicians, the immediate takeaway is to shift from a paternalistic stance of warning patients away from the internet to a collaborative one. This involves routinely and nonjudgmentally assessing online information-seeking behaviors; using the digital phenotype concept to gauge readiness for change; and explicitly addressing the information void by setting realistic expectations for biomarker improvement and providing proximate, behavior-linked goals. For health systems and policymakers, our analysis provides a strong rationale for investing in scalable digital health literacy programs and exploring new reimbursement models that compensate for digital education. Furthermore, it highlights the need for clear institutional guidelines on professional boundaries and for policy discussions on the responsibilities of social media platforms in curating a healthier information environment.

Our analysis is characterized by several strengths, including its novel synthesis of infodemiology, behavioral science, and digital health research into a cohesive and actionable framework supported by established theoretical models. By triangulating evidence from diverse sources and grounding the framework in recognized theories (COM-B, temporal discounting, and uses and gratifications theory), we provide a comprehensive perspective on a complex problem. The inclusion of illustrative case studies demonstrates the framework’s practical application and clinical relevance. Nevertheless, we acknowledge important limitations. As a viewpoint, this work is conceptual and does not present new primary data. The 2×2 framework deliberately simplifies a complex reality; individual patients’ vulnerability is modulated by numerous factors beyond disease characteristics, including cultural context, health system structure, socioeconomic status, digital access, and individual health literacy [27,28]. The framework has been developed primarily with reference to Western health care contexts and social media platforms; its applicability to different cultural settings, health care systems, and digital ecosystems (eg, WeChat-dominated health information environments in China or settings with different patient-health care provider dynamics) requires empirical validation [20]. The digital landscape is in constant flux, meaning that platform-specific findings may quickly become outdated. Moreover, the digital divide remains a critical challenge; interventions that rely on digital access and literacy risk exacerbating existing health disparities if not designed with equity at their core. The evidence base for interventions, while strong for intermediate outcomes such as literacy, requires more research linking these interventions to hard clinical end points.

## Conclusions

The intersection of rising asymptomatic chronic diseases in young adults and their universal engagement with social media has created a critical public health challenge. The information void that characterizes these conditions, grounded in the convergence of absent somatic feedback and delayed clinical feedback, makes this population uniquely vulnerable to digital misinformation. However, this challenge also presents an opportunity. By reframing online information seeking as a digital phenotype, we can transform a perceived problem into a powerful tool for patient engagement. The evidence clearly points toward a constructive path forward: a sustained, multipronged strategy that builds digital health literacy, delivers high-quality curated resources, addresses the algorithmic structures that amplify misinformation, and fosters strategic institutional engagement. Future research must now rise to the challenge of validating this framework through prospective studies across diverse health care settings and populations; designing and testing interventions that directly address the psychological drivers of this behavior; and developing novel methods to evaluate their effectiveness in the complex, dynamic digital ecosystem.

## Abbreviations

COM-B: capability, opportunity, and motivation–behavior
